# Motivational Processes Influencing Mental Health Among Winter Sports Athletes in China

**DOI:** 10.3389/fpsyg.2021.726072

**Published:** 2021-09-17

**Authors:** Xinran Wu, Nor Eeza Zainal Abidin, Rafidah Aga Mohd Jaladin

**Affiliations:** ^1^Center for Sport and Exercise Sciences, University of Malaya, Kuala Lumpur, Malaysia; ^2^Department of Educational Psychology and Counseling, Faculty of Education, University of Malaya, Kuala Lumpur, Malaysia

**Keywords:** motivational processes, psychological distress, burnout, self-determination theory, modeling

## Abstract

This study examined the association between motivational processes, psychological distress (depression, anxiety, and stress), and burnout among winter sports athletes within the Hierarchical Model of Intrinsic and Extrinsic Motivation (HMIEM). A total of 685 winter sport athletes participated in this study (377 males, 308 females, age range 18–25 years), from three sport universities across nine winter sports. They completed five psychometric inventories related to motivational factors and mental disorders. Overall, a task-oriented climate showed a positive association with basic psychological needs, eliciting a positive pathway to autonomous and controlled motivation. In contrast, an ego-oriented climate showed a negative association with basic psychological needs, eliciting a negative pathway to amotivation. Autonomous and controlled motivation were negatively associated with symptoms of psychological distress and burnout, while amotivation was positively associated with symptoms of psychological distress and burnout. These findings highlight the complex relationships between various motivational factors and mental health disorders among winter sport athletes, and support the essential requirement for adding mental health factors to the outcomes of the HMIEM sequence.

## Introduction

Mental health is vital for athletes' overall health. Athletes are pressured to be physically superior and perform both in front of opponents and spectators alike. Over time, these athletes become more concerned about their strong physical appearance and athletic performance, and they tend to place less emphasis on, or even ignore their mental health needs. Furthermore, a previous study showed that serotonin levels, which are associated with happiness, in the body are markedly decreased while the chemical associated with mental health crises are increased during winter months (Gupta et al., 2013). This highlights the need to conduct a study on mental health among winter sports athletes, especially in view of the upcoming 2022 Winter Olympics that will be held in China.

In the present research, psychological distress was conceptualized as an overarching construct representing negative states that may affect athletes' mental health such as depression, anxiety, and stress (Reardon et al., 2019). In addition, three relevant factors of athlete burnout, which included exhaustion, sport devaluation, and reduced sense of personal accomplishment, were used to measure the level of burnout among winter sports athletes (Raedeke and Smith, 2001).

Longstanding evidence supports that motivation is an important predictor of mental health (Deci and Ryan, 2000). Furthermore, previous studies in sports were mainly focused on summer sports, such as golf, rugby, basketball, swimming, and football (Barcza-Renner et al., 2016; Schaefer et al., 2016; Sheehan et al., 2018; Vella et al., 2020). Unfortunately, there are not many studies available which have linked mental health to motivation in winter sports. Therefore, the main purpose of the current study was to examine winter sports athletes' psychological distress and burnout in the motivational processes within the Hierarchical Model of Intrinsic and Extrinsic Motivation (HMIEM) (Vallerand, 1997). This study provides practical applications for enhancing the motivation and mental health of winter sports athletes.

### Theoretical Integration of SDT and HMIEM

The Self-Determination Theory (SDT) is a broad framework for the study of human motivation (Deci and Ryan, 2000). It is an important concept that refers to people's ability to make choices and manage their behavior, and this ability plays an important role in mental health. According to SDT, there are primary forms of motivation, intrinsic and extrinsic, which have a significant influence on how people act (Deci and Ryan, 2000). Extrinsic motivation is derived from external factors, while intrinsic motivation is derived from internal factors, and each type of motivation has a different effect on human behavior (Di Domenico and Ryan, 2017). Intrinsic motivation is the most self-determined modality in terms of satisfaction and enjoyment. For example, we are intrinsically motivated to participate in a given exercise because we are fond of it and get personal satisfaction from it.

Extrinsic motivation is considered to be a behavior that is driven by external incentives, such as grades, praise, fame, and other material rewards. This form of motivation is comprised of four types of regulation: integrated regulation is a form of extrinsic motivation which is the most autonomous, where people participate in an activity that is of personal importance to them and aligns to their personal values (e.g., a skier who continues training because that behavior aligns with his values system, even if he doesn't have an enjoyable feeling during the training.); identified regulation is a form of extrinsic motivation where individuals are free to choose unenjoyable behaviors in order to achieve meaningful results (e.g., a skater who recognizes that hard training is important for becoming a successful athlete.); introjected regulation is a form of extrinsic motivation where people engage in activities, because they feel they should in order to avoid feeling guilty or ashamed, or to maintain their sense of self-worth (e.g., a curling player who persist with his training because he fears a negative reaction from his peers if he does not behave like that.); and external regulation, the purest form of extrinsic motivation, where individuals perform behaviors in order to obtain a reward or avoid punishment (e.g., an ice hockey player who continues training hard in order to obtain praise or avoid punishment from his coach or team leader).

While extrinsic and intrinsic motivation are usually recognized as separate, the reasoning behind a given behavior is often complicated, and people are seldom driven to act by a single source of motivation. When people are in pursuit of a goal, they often draw on multiple types of motivation. For example, in the case of a person who is training to compete in skiing, they might be intrinsically motivated by the satisfaction achieved from the activity of skiing itself as well as extrinsically motivated by a desire to earn approval from coaches or parents. Overall, autonomous motivation incorporates intrinsic motivation, identified regulation, and integrated regulation, which come from internal and external resources for athletes who identify with a value of behavior and how it aligns with their self-worth. On the other hand, controlled motivation includes introjected and external regulations, which stem from external resources. Amotivation represents a lack of intention to participate in an activity, characterized by a lack of perceived ability or a failure to value the behavior and its results. At present, SDT is the most widely accepted theory in the motivation of competitive sport (Clancy et al., 2016), and is a highly appropriate conceptual framework from which to understand sport motivation.

HMIEM is an extension of the SDT, which posits that a thorough analysis of motivation outcomes (affect, cognition, and behavior) is needed in order to consider the whole motivational process (Vallerand, 1997). This model addresses the determinants and outcomes related to the different types of motivation at situational, contextual, and global levels. The most abstract level of the three levels is the global level, which considers an individual's personality or usual way of engaging in life activities in a typically intrinsic or extrinsic way. Following the global level is the contextual level, which is the least abstract level within the HMIEM model. It depicts specific life contexts, such as work, sport, and education. This level indicates the possibility that an individual may develop a different motivational orientation within the individual under different contexts. For example, an individual may learn history in a more internal way, but participate in sports with a greater emphasis on external motivation. Therefore, in order to make accurate predictions about motivations and consequences, it is vital to consider the type of activities people are engaged in. Finally, the most specific level is the situational level, which refers to the motivation of the individual in the specific moment. For instance, someone who is diligently training at skiing at 4 o'clock on Friday morning is operating with intrinsic motivation. This is the state of motivation that a person experiences when participating in an activity at a specific time (Vallerand and Lalande, 2011).

The HMIEM model is a powerful argument in favor of motivational processes that include four stages. In short, HMIEM includes the following motivational process sequence: Social Factors → Basic Psychological Needs → Motivation → Consequences. The current research predominantly focuses on the above-mentioned motivational sequence at the contextual level because individual motivational behavior is included in this level in a particular life area, such as sport.

### Determinants of Motivation in Sport

HMIEM takes into account social factors because they are assumed to have a profound effect on athletes' motivation. In this model, the social-environment factor is called the motivational climate, and the framework of motivational climate is developed from the Achievement Goal Theory (AGT) (Ames, 1992). Regarding specific viewpoints of AGT, the motivational climate is affected by the teammates, coaches, leaders, or sport structures. Although many factors in the context of sport may have an influence on athletes 'motivation, such as scholarships or financial bonuses, the perception of coaching behavior is regarded as one of the most crucial factors within the sport motivational climate (Amorose, 2007). The AGT was used as a guiding framework and has been largely concerned with two different factors: task-oriented climate (also known as mastery orientation), and ego-oriented climate (also known as performance orientation) (Duda and Balaguer, 2007). In sport research, the AGT is the most widely used theory in the conceptualization of motivational climate (Lindahl et al., 2015). Perceptions of a task-oriented climate were connected with outcomes deemed to be more vigorous, such as hard training, increased competence, and skill improvement. In contrast, perceptions of an ego were related to less positive results and more maladaptive motivation, such as amotivation, training avoidance, and trait anxiety. Furthermore, previous research demonstrated that the task-oriented climate can develop the levels of satisfaction, enjoyment, and overall interests of athletes in participating in sports, whereas an ego-oriented climate tends to increase stress, tension, and performance anxiety (Harwood et al., 2015).

The concept of basic psychological needs is a crucial factor within the construct of HMIEM, as motivation is influenced by the motivational climate's impact on basic psychological needs (Vallerand, 1997). According to Ryan and Deci, there are three forms of basic psychological needs, which are innate rather than acquired human tendencies: autonomy, competence, and relatedness (Ryan and Deci, 2000). Autonomy represents the feeling that people need to feel that they are in control of their behavior. Competence refers to building their capability and improving their mastery over tasks that are crucial to them. Finally, relatedness indicates that people need to have a sense of connection and belonging with others (Deci and Ryan, 2008). Within a task-oriented climate, the coach likes to offer enough choices (autonomy support) to their athletes, trust in the abilities of their athletes (competence support), and takes their feeling into consideration (relatedness support). In contrast, within an ego-oriented climate, a coach's behavior does not support basic psychological needs because they often use pressure and control to influence the athlete's behavior (Harwood et al., 2015). According to Vallerand, these basic psychological needs are the critical link between social environment and human motivation in the HMIEM.

### Motivational Outcomes

On the importance of motivation as a whole, much evidence has shown that motivational research is well-established across numerous domains, including education, business, medicine, and physical activity. As such, it has been demonstrated that it is important to find ways to boost motivation because it helps individuals to improve habits (Gardner and Rebar, 2019), be creative (Bhakti et al., 2018), boost engagement (Yun et al., 2020), and emphasize physical rehabilitation (Maclean et al., 2000). SDT claims that the more self-determined the motivation, the more favorable the results (Vallerand, 1997). In sports, self-determining motivation is linked to a series of results, such as dropout (Pelletier et al., 2001), effort (Pope and Wilson, 2012), engagement (Podlog et al., 2015), persistence (Rottensteiner et al., 2015), enjoyment (Russell et al., 2017), burnout (Fagundes et al., 2021), perceived performance (Almagro et al., 2020), and mental health (Sheehan et al., 2018). These studies supported that the direct or indirect relationships between motivation and mental health during physical exercise are of great importance, and this complex association continues to arouse the interest of researchers.

On the one hand, elite athletes experience various specific pressures during their individualized sporting competitions. However, they also need to contend with the training strategy of coaches and the relationships with their teammates. As a whole, sport participation alone cannot reduce the incidence of mental disorders when it comes to elite athletes. What is more serious and relevant to this current study is the fact that as an athlete's symptoms of mental disorders intensify, their performance may be affected, thus making them frightened and further exposed to additional signs and further symptoms of common mental disorders (Souter et al., 2018). It is worth noting that these mental disorders have been related to various elements of the HMIEM model, such as motivational climate were related to anxiety and fear of failure (Gómez-López et al., 2020), basic psychological needs were related to life satisfaction and burnout (Chen et al., 2015; Kent et al., 2018), and various types of motivation were related to anxiety, depression, mood disturbance, and sleep quality (Sheehan et al., 2018). Therefore, according to available research, studies demonstrate that mental health is capable of playing a part in the motivational process of HMIEM.

### Motivational Processes and Mental Health

The research has shown that anxiety, depression, stress, and burnout still occur among athletes, and most stakeholders are interested in these mental symptoms because they affect the performance of athletes. Previous studies support that these mental disorders have been linked to various components of the HMIEM model. One of the recent studies reported that higher levels of anxiety were linked to an ego-oriented climate, while a task-oriented climate was related to lower levels of anxiety (Castro-Sánchez et al., 2019). The satisfaction of basic psychological needs is considered a mediator between motivational climate and motivation (Deci and Ryan, 2000). Notable, basic psychological needs have also been found to be a robust predictor of a variety of mental health indicators, including burnout components (Morano et al., 2020), depression (Cordeiro et al., 2016), anxiety (Haraldsen et al., 2020), and stress (Li et al., 2019). Furthermore, there has been previous evidence for an association between motivation and various types of mental health (Stenling et al., 2017; Sheehan et al., 2018), specifically when testing for depression or anxiety as part of the HMIEM. Overall, the HMIEM is a potentially useful model for comprehending the depression, anxiety, stress, and burnout of winter sport athletes, as these mental disorders are directly associated with athletic performance. The present research focuses on the expansion of existing theories, and provides suggestions for maintaining mental health through motivational processes in sport.

## The Present Study

The purpose of this present research was to test the motivational sequence of the four-stage in the realm of sport within the HMIEM. In particular, the objective of the current research was to adopt structural equation modeling to incorporate athletes' mental disorders into motivational processes within the HMIEM: motivational climate in sport → basic psychological needs in sport → sport motivation → athletes' mental disorders. Our hypotheses are based on the HMIEM and related SDT literature, which are: (1) an ego-oriented climate would have a negative association with basic psychological needs; a task-oriented climate would have a positive association with basic psychological needs. (2) basic psychological needs would have a negative association with amotivation; basic psychological needs would have a positive association with autonomous motivation; basic psychological needs would have a positive association or have no association with controlled motivation. (3) amotivation would have a positive association with psychological distress and burnout; autonomous motivation would have a negative association with psychological distress and burnout; controlled motivation would have a positive association or have no association with psychological distress and burnout.

## Methods

### Participants

A total of 685 college winter sports athletes (55% male, 45% female) participated in this study. Table 1 showed their gender, age, and training characteristics. They came from three sport universities across 9 winter sports, including alpine skiing (*N* = 167, 24.4%), ice hockey (*N* = 152, 22.2%), speed skating (*N* = 122, 17.8%), curling (*N* = 85, 12.4%), figure skating (*N* = 78, 11.4%), snowboarding (*N* = 46, 6.7%), freestyling (*N* = 15, 2.2%), biathlon (*N* = 11, 1.6%), and cross-country skiing (*N* = 9, 1.3%). Participants ranged in age from 18 to 25 (M = 20.5, SD = 1.5), and 86.6% of them had more than 5 years of training experience. Of the athletes, 41.6% had a low competence level (national second-level), 46.4% had a medium competence level (national first-level), 9.0% had a high competence level (national master-level), and 3.0% had a top competence level (international master-level). Of the total, 58.8% were individual event athletes and 41.2% were team event athletes. 33.0% of participants trained for 2–3 sessions per week, and 33.6% of participants trained 4–5 sessions per week, while every participant engaged in training consisting of an average duration of 120 min/session.

**Table 1:** Gender, age and training characteristics of the sample of winter sports athletes analyzed in the study.

	**Male (*n* = 377)**	**Female (*n* = 308)**	**Total (*n* = 685)**
Age			
18–19 20–21 22–23 24–25	105 (27.9%) 158 (41.9%) 63 (16.7%) 51 (13.5%)	88 (28.6%) 138 (44.8%) 59 (19.1%) 23 (7.5%)	193 (28.2%) 296 (43.2%) 122 (17.8%) 74 (10.8%)
Training time			
<5 years 5–7 years 8–10 years >10 years	38 (10.1%) 169 (44.8%) 152 (40.3%) 18 (4.8%)	54 (17.5%) 119 (38.6%) 104 (33.8%) 31 (10.1%)	92 (13.4%) 288 (42.0%) 256 (37.4%) 49 (7.2%)
Training volume (120 min per session)			
1 session/ week 2–3 sessions/ week 4–5 sessions/ week 6 sessions/ week	73 (19.4%) 119 (31.6%) 125 (33.1%) 60 (15.9%)	63 (20.5%) 107 (34.7%) 105 (34.1%) 33 (10.7%)	136 (19.8%) 226 (33.0%) 230 (33.6%) 93 (13.6%)
Sport modality			
Individual Team	232 (61.5%) 145 (38.5%)	171 (56%) 137 (44%)	403 (58.8%) 282 (41.2%)
Level of competition			
National second-level National first-level National master-level International master-level	178 (47.2%) 166 (44.0%) 27 (7.2%) 6 (1.6%)	107 (34.7%) 152 (49.4%) 35 (11.4%) 14 (4.5%)	285 (41.6%) 318 (46.4%) 62 (9.0%) 20 (3.0%)
Sport items			
Alpine skiing Ice hockey Speed skating Curling Figure skating Snowboard Freestyling Biathlon Cross-country skiing	93 (24.7%) 102 (27.0%) 66 (17.5%) 32 (8.5%) 31 (8.2%) 29 (7.7%) 11 (2.9%) 7 (1.9%) 6 (1.6%)	74 (24.0%) 50 (16.2%) 56 (18.2%) 53 (17.2%) 47 (15.3%) 17 (5.5%) 4 (1.3%) 4 (1.3%) 3 (1.0%)	167 (24.4%) 152 (22.2%) 122 (17.8%) 85 (12.4%) 78 (11.4%) 46 (6.7%) 15 (2.2%) 11 (1.6%) 9 (1.3%)

### Data Collection Procedure

After being granted approval from the Ethics Committee from the researchers' institution and obtained permission for conducting surveys from the respective universities in northeastern China, data collection phase commenced. The researchers approached participants' institutional leaders and team coaches first before handing out the survey questionnaires to the selected participants. Participants completed the pen-and-paper surveys in the absence of their coaches at their respective sporting club, aided by a research assistant. Prior to the distribution and completion of questionnaires, the researchers provided standardized written and verbal instructions to participants to enable them to better understand the meaning of the questions and manage their answering time. Participants answered five questionnaires, and took approximately 25 minutes to complete. Data distribution and collection took place from the middle to the end of the winter sports season.

### Measures

#### Perceived Motivational Climate in Sport Questionnaire-2

Athletes completed a 33-item questionnaire designed to assess perceptions of their team was using the Perceived Motivational Climate in Sport Questionnaire-2 (PMCSQ-2; Newton et al., 2000). The PMCSQ-2 assigns scores on the perceived task and ego-oriented climates, with the following stem (sample item: “on this team, the coach gets mad when a player makes a mistake”; “on this team, players feel good when they try their best”). This subscale was measured on a five-point Likert scale. Cronbach's alpha coefficients of the task-oriented (0.88) and ego-oriented climates (0.87) were acceptable for each (Newton et al., 2000). According to the meta-analysis by Harwood et al. (2015), the PMCSQ-2 is the most widely used measurement for the assessment of motivational climate.

#### Basic Need Satisfaction in Sport Scale

Athletes completed a 20-item scale, the Basic Need Satisfaction in Sport Scale (BNSSS; Ng et al., 2011), which is designed to assess perceptions of their competence, autonomy, and relatedness. This subscale asks athletes how the athletes feel when they are participating in sports (sample item: “in my sport, I feel I am pursuing goals that are my own”) and it was measured on a seven-point Likert scale. Cronbach's alpha of the scale between 0.61 and 0.86 for the five subscales as considered reliable with range (Ng et al., 2011).

#### Sport Motivation Scale-2

Athletes completed the 18-item Sport Motivation Scale-2 (SMS-2; Pelletier et al., 2013) to assess motivation in line with the HMIEM model. The SMS-2 provides questions for athletes to answer concerning why they practice sports (sample item: “because it is very interesting to learn how I can improve”). This subscale was measured on a seven-point Likert. Cronbach's alpha of the scale between 0.73 and 0.86 was considered reliable with range (Pelletier et al., 2013). The SMS-2 subscales were considered a valid tool for sports motivation.

#### Depression, Anxiety and Stress Scale (DASS-21)

The Depression, Anxiety and Stress Scale−21 Items (DASS-21) is a series of three self-report scales designed to assess the emotional states of anxiety, depression, and stress (Lovibond and Lovibond, 1995). This subscale was measured on a four-point Likert scale. Cronbach's alpha values of 0.82, 0.83, and 0.80 for the subscales of stress, depression, and anxiety, respectively (Wang et al., 2016). DASS-21 shows good reliability and is widely used in mental health measurement.

#### Athlete Burnout Questionnaire

The Athlete Burnout Questionnaire (ABQ) is a 15-item questionnaire that presents three dimensions: emotional/physical exhaustion, reduced sense of accomplishment, and sport devaluation (Raedeke and Smith, 2001). This subscale asks athletes how they feel when they are participating in sport, with the following stem (sample item: “I feel extremely tired from the sport participation”) and was measured on a five-point Likert scale. Cronbach's alpha of the scale of 0.92, 0.86, and 0.92 for the three dimensions were considered reliable with range (Raedeke and Smith, 2001).

### Translation Procedures

Due to the fact that the majority of the measures in the survey were originally developed in English, the questionnaires were translated and validated in Chinese before the data were collected. Although the PMCSQ-2 (Cai, 2016), BNSSS (Ng, 2008), SMS-2 (Li et al., 2016), DASS-21 (Gong et al., 2010), and ABQ (Chen and Zhou, 2007) had already been translated and validated in Chinese speaking samples, we included them in the translation process. Translation and back-translation of five questionnaires have been done by two academic language experts who are fluent in both English and Chinese. To verify the validity of scales, the Chinese version of the questionnaires were submitted to three experts, and modifications were made on the basis of feedback obtained from them.

### Data Analysis

**Figure 1:**
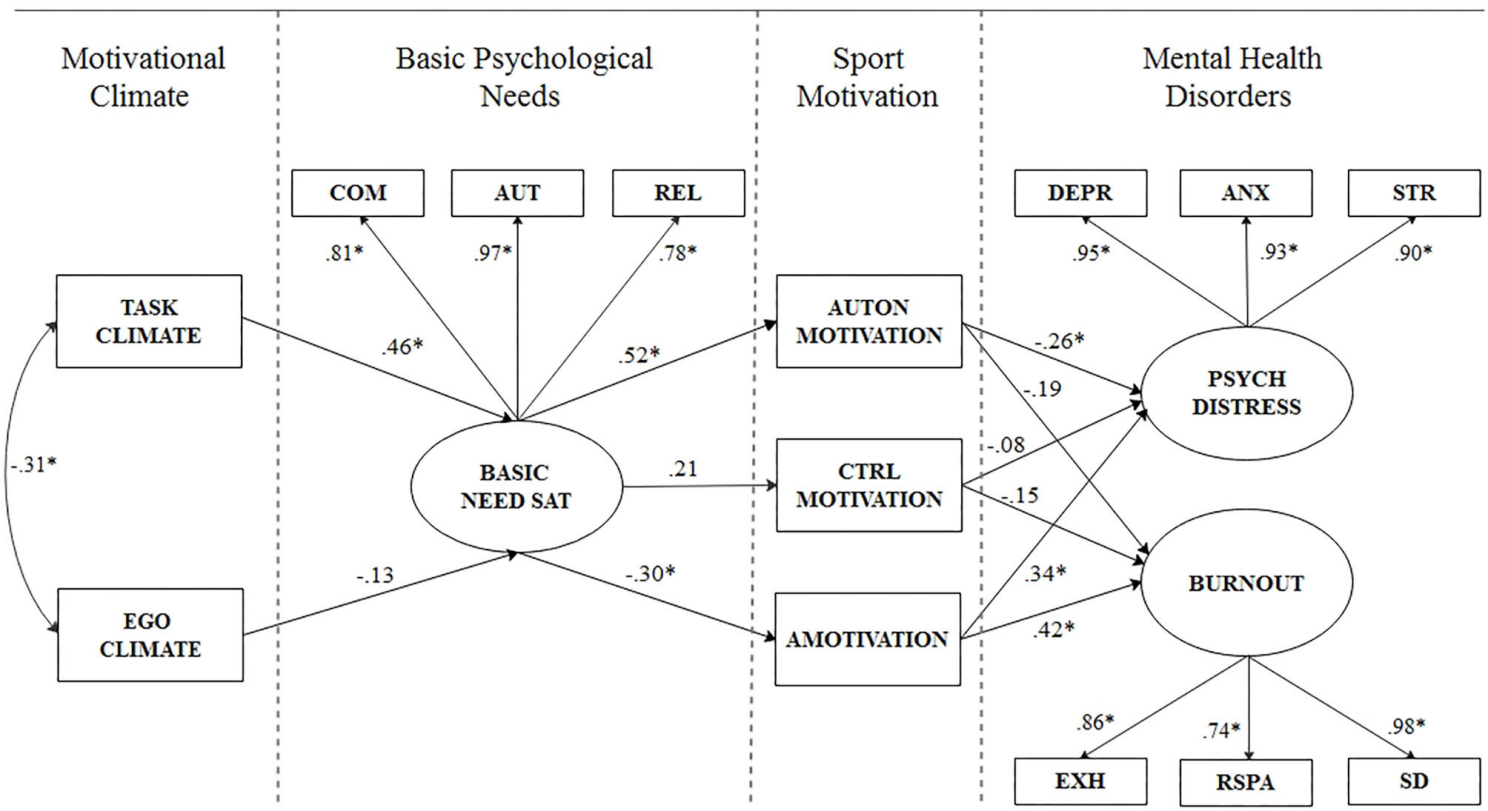
Standardized path coefficients from the SDT structural models. BASIC NEED SAT, basic needs satisfication; PSYCH DISTRESS, psychological distress; COM, competence; AUT, autonomy; REL, relatedness; AUTON, autonomous; CTRL, controlled; DEPR, depression; ANX, anxiety; STR, stress; EXH, exhaustion; RSPA, reduce sense of personal accomplishment; SD, sport devaluation.

To analyze the research data, SPSS Statistics 26.0 and Amos 26.0 were used in this research, and descriptive statistics were used to summarize the athletes' scores of lists. The correlation coefficients of Pearson were used to measure the relationships between variables: 0.50 is large, 0.30 is moderate, and 0.10 is small (Cohen, 1988). To examine internal consistency, Cronbach's alpha coefficients were calculated, and the acceptable cut-off score was determined to be 0.70 (Nunnally, 1978). Finally, structural equation modeling was used to evaluate and specify a conceptual model (Figure 1) describing the four-stage integrated sequence within HMIEM. CFI and TLI values >0.90 and 0.95 are usually considered to indicate reasonable and excellent fit, while RMSEA and SRMR values <0.08 indicate the boundaries of acceptable fit (Hu and Bentler, 1999). AMOS was used to complete all SEM analyses in this investigation. In further testing the SEM, we used a standardized regression coefficient to quantify the association between variables and interpreted results according to effect size criteria: 0.50 is large, 0.30 is moderate, and 0.10 is small (Cohen, 1992).

## Results

### Preliminary Analysis

The result shows that 31% of subjects suffered from varying degrees of psychological distress. On average, the college winter sports athletes had low depressive, anxiety, and stress symptoms. When categorized as specific symptoms, 21.0% had mild to moderate depressive symptoms (23.6% males, 17.9% females), 6.0% had severe depressive symptoms (5.8% males, 5.8% females), 9.1% had mild to moderate anxiety disorders (10.1% males, 7.8% females), 6.9% had severe anxiety disorders (8.0% males, 5.5% females), 10% had mild to moderate stress disorder (9.0% males, 11.4% females), and 5.0% had severe stress disorder (5.0% males, 4.9% females). Additionally, 15.9 % had moderate to severe burnout symptoms (18.0% males, 13.3% females).

### Descriptive Statistics and Correlations

The means and standard deviations of the variables in this study are shown in Table 2. The motivational climates were measured by the Perceived Motivational Climate in Sport Questionnaire, with the task-oriented climate in sport presenting the highest mean (M = 4.35), whereas the lowest mean referred to the ego-oriented climate in sport (M = 2.93). Basic Need Satisfaction in Sport Scale was used to measure the basic psychological needs, with relatedness presenting the highest mean (M = 5.71), competence presenting the second highest mean (M = 5.47), and autonomy presenting the lowest mean (M = 5.11). Additionally, we used the Sport Motivation Scale to measure motivation, with controlled motivation (M = 3.25) and amotivation (M = 2.55) presenting with a lower mean than autonomous motivation (M = 5.56).

**Table 2:** Descriptive statistics and bivariate correlations between study variables.

			**Correlations among scales**													
**Variables**	**M**	**SD**	**1**	**2**	**3**	**4**	**5**	**6**	**7**	**8**	**9**	**10**	**11**	**12**	**13**	**14**
Task climate	4.35	0.76	–													
Ego Climate	2.93	1.05	**−0.31**	–												
Competence	5.47	1.34	**0.48**	**−0.26**	–											
Autonomy	5.11	1.29	**0.50**	**−0.27**	**0.76**	–										
Relatedness	5.71	1.29	**0.47**	**−0.20**	**0.61**	**0.64**	–									
Autonomous Motivation	5.56	1.34	**0.50**	**−0.32**	**0.29**	**0.34**	**0.27**	–								
Controlled Motivation	3.25	1.25	**0.11**	0.04	**0.16**	0.09	**0.17**	**0.28**	–							
Amotivation	2.55	1.74	**−0.34**	**0.38**	**−0.13**	**−0.19**	**−0.15**	**−0.46**	**0.35**	–						
Depression	0.99	1.24	**−0.43**	**0.35**	**−0.25**	**−0.30**	**−0.23**	**−0.49**	−0.02	**0.53**	–					
Anxiety	0.81	1.10	**−0.32**	**0.25**	**−0.22**	**−0.29**	**−0.22**	**−0.35**	**–**0.03	**0.37**	**0.74**	**–**				
Stress	0.88	1.25	**−0.33**	**0.24**	**−0.17**	**−0.21**	**−0.18**	**−0.38**	**–**0.05	**0.36**	**0.70**	**0.74**	**–**			
Exhaustion	1.81	0.84	**−0.31**	**0.23**	**–**0.09	**−1.35**	**–**0.08	**−0.27**	**–**0.03	**0.35**	**0.45**	**0.39**	**0.43**	–		
RSPA	2.43	0.84	**−0.24**	**0.24**	**−0.13**	**–**0.05	**–**0.06	**−0.34**	**−0.13**	**0.32**	**0.32**	**0.17**	**0.29**	**0.53**	–	
Sport Devaluation	1.92	0.96	**−0.34**	**0.30**	**−0.19**	**−0.22**	**−0.17**	**−0.40**	**–**0.09	**0.45**	**0.53**	**0.41**	**0.47**	**0.66**	**0.57**	–

*RSPA, Reduced Sense of Personal Accomplishment; Correlations significant at p < 0.05 have been bolded*.

It can also be observed in Table 2 that most of the Pearson correlations between factors had statistically significant values. The strongest relationship was observed between autonomy and competence (β = 0.76, P < 0.01), and the weakest relationship was observed between controlled motivation and task-oriented climate (β = 0.11, P < 0.05).

### Reliability of the Scales

Cronbach's alpha coefficient for the SMS-2 subscales ranged from 0.81 to 0.96, autonomous motivation (a = 0.92), controlled motivation (a = 0.81), and amotivation (a = 0.86); task-oriented climate (a = 0.96) and ego-oriented climate (a = 0.93); competence (a = 0.93), autonomy (a = 0.89), and relatedness (a = 0.85); depression (a = 0.84), anxiety (a = 0.85), stress (a = 0.85), and burnout (a = 0.85), with all values above the threshold of 0.70.

### SEM Model

In conjunction with the formulate hypotheses, a model was specified with basic needs satisfaction, psychological distress, and burnout as three latent variables, and five manifest variables, including task-oriented climate, ego-oriented climate, autonomous motivation, controlled motivation, and amotivation. The SDT-based model including mental disorders outcomes showed acceptable fit to the data (Figure 1): χ2 = 239 (*p* < 0.001), df = 66, CFI = 0.92, TLI = 0.89, RMSEA = 0.07, 90% confidence interval [0.05, 0.09], SRMR = 0.04.

### Hypothesis 1

A task-oriented climate had a moderate to large positive association (β = 0.46) with basic psychological needs, and an ego-oriented climate had a small negative association (β = -0.13) with basic psychological needs.

### Hypothesis 2

Basic psychological needs had a large positive association (β = 0.52) with autonomous motivation, a small to moderate positive association (β = 0.21) with controlled motivation, and a moderate negative association (β = -0.30) with a motivation.

### Hypothesis 3

Autonomous motivation had a small to moderate negative association (β = -0.26) with psychological distress, and a small negative association (β = -0.19) with burnout. Controlled motivation had a small negative association with psychological distress (β = -0.08) and burnout (β = -0.15). Amotivation had a moderate positive association (β = 0.34) with psychological distress and a moderate to large positive association (β = 0.42) with burnout.

## Discussion

The objective of the present study was to evaluate a sequence of motivational processes originally proposed by Vallerand and extending previous motivational research. Specifically, the main purpose of the current research was to test a model with hypothesized associations between motivational climate, basic psychological needs, sport motivation, psychological distress, and burnout among Chinese winter sport athletes within the HMIEM model. Overall, the athletes reported high level (above the midpoint) of task-oriented climate, basic need satisfaction, and autonomous motivation, and low level (below the midpoint) of psychological distress and burnout. The findings of this research showed that depression, anxiety, stress, and burnout can be components of the motivational sequence in HMIEM. It is reasonable to conclude that athletes' motivational processes play an important role in mental health maintenance. This study reveals the relationship between three original factors in HMIEM and mental disorders variables, filling a gap in Chinese research in this area by investigating Chinese winter sports athletes. Importantly, there are not many studies on motivation and mental health of winter sports athletes, and this research may have significant implications for improving their overall motivation and mental health.

### Motivational Climate and Basic Psychological Needs

The outcomes of this research provided substantial support for the proposed model. With respect to the first part of the hypothesized sequence, the outcomes indicated college winter sports athletes' perceptions of a task-oriented climate to be positively related to their basic need satisfaction of the needs concerning competence, autonomy, and relatedness. The observed positive association between task-oriented climate and basic psychological needs is consonant with previous research (Reinboth and Duda, 2006). In particular, a coach who creates a task-oriented climate will instill beliefs in athletes, respect athletes' autonomy, and maintain a good relationship with athletes, which leads to the satisfaction of basic needs. On the contrary, an ego-oriented climate had a negative association with basic need satisfaction, as similarly indicated in a previous study (García-González et al., 2019), Clearly, when college winter sport athletes train in an atmosphere that emphasizes intra-team competition, comparing themselves to each other and recognizing only the most capable athletes, it is likely to reduce the sense of belonging and intimacy among team members. Given these findings, this means that an ego-oriented climate on a team may not be appropriate for college winter sport athletes who need to be encouraged, appreciated, and accepted by those whose views are particularly important. However, this result differs from the findings of Reinboth and Duda (2006), who found that the ego-oriented climate was not associated with the basic need satisfaction of competence and autonomy. The differences in the findings may be due to the different respondents. High level athletes were more confident than average athletes, and they may be able to get more autonomy from their coach. As such, when they are engaging in a more ego-oriented climate, this would not be expected to have a negative effect, or possibly even no impact, on basic psychological needs. It can be understood that the level of perceived ability may play a moderating role in the relationship between ego-oriented climate and basic psychological needs.

### Basic Psychological Needs and Motivation

In the second part of this study, based on the sequence assumed in the Vallerand (2007), the expected interaction between the satisfaction of basic psychological needs and more self-determined reasons for participation were verified, and the results showed that the basic psychological needs were significantly and positively associated with autonomous motivation. This finding is in line with previous research revealing that basic psychological needs are important predictors of autonomous motivation (e.g., Hollembeak and Amorose, 2005). Secondly, the positive effect of basic psychological needs on controlled motivation was relatively modest by comparison. According to the developers of SDT (Deci and Ryan, 2008), all three basic needs have a positive impact on autonomous motivation, with only competence and relatedness have a positive effect on controlled motivation, while autonomy does not. As such, this may be the reason why the basic psychological needs have a greater positive effect on autonomous motivation than on controlled motivation, while others have reported a negative relationship between basic psychological needs and controlled motivation (Bartholomew et al., 2018). Finally, it is unsurprising that the observed negative association between basic psychological needs and amotivation may be reflecting discouragement, helplessness, and disengagement, as this finding is consonant with previous research (Haerens et al., 2015; Jang et al., 2016; Bartholomew et al., 2018).

### Motivation and Mental Health Disorders

In the third part of this study, the findings show that both autonomous and controlled motivation were negatively associated with psychological distress and burnout, which is somewhat different from previous studies. There is no denying that considerable research in sport that demonstrates a strong positive association between autonomous motivation and some positive outcomes, such as effort, persistence, good performance, and high level of psychological well-being (Vallerand, 2007). On the other hand, much evidence supports that controlled motivation had a positive association with mental disorders, such as burnout (Jowett et al., 2013) as well as mood disturbance and anxiety (Sheehan et al., 2018). In the present study, this was not the case among the athletes, as controlled incentives, had a negative association with mental disorders. Although autonomous incentives seem to be more positively associated with mental health in sport, no doubt, this does not mean that we should abandon external rewards. Controlled motivation is a good performer in its own right. When used properly, it can still have a positive effect on mental health.

Furthermore, winter sports have their own special characteristics. For college athletes, not only it is generally colder and the days are shorter, but the dual-pressure of athletic competitions and academic exams by this time of the year without much rest. It is not uncommon for college winter sport athletes to wake up when it is dark, then go to training, only to not return back home until it is dark again. These factors may be contributing to the lack of motivation for college athletes to train. With these in mind, for winter sports athletes, controlled incentives are as important as autonomous incentives when it comes to boosting motivation. It is possible that the Chinese college winter sports athletes were conditioned by controlled motivation for a long period of time. When they have become accustomed to this way and have achieved good competitive results or satisfaction with training performance, this approach may have a positive impact on mental health. Therefore, a negative relationship between controlled motivation and mental health disorders was found in this research, and it is worth noting that the satisfaction of competition performance or training performance may act as a mediating variable between controlled motivation and mental health.

Finally, it is unsurprising that the observed positive relationship between amotivation and mental health disorders is consistent with the main principle of SDT (Deci and Ryan, 2008). According to the developers of HMIEM (Vallerand, 1997), psychological maladjustment may occur when athletes lack intentionality about their act. On the other hand, lack of motivation or diminished drive to complete goal-directed activities is a common and concerning characteristic in people with mental disorders (Remington et al., 2016). In a similar study, amotivation was found to be associated with burnout in a survey of NCAA Division 1 swimmers (Barcza-Renner et al., 2016). In conclusion, most of the relationships between motivational processes and the outcomes of mental disorders in present study were consistent with previous studies and theories.

### Practical Implications

This is a study examining the association between motivational processes, psychological distress (depression, anxiety, and stress), and burnout among winter sports athletes in HMIEM. Therefore, it has made an important contribution to the study of motivation and mental disorders, revealing how motivational processes influence mental health, particularly where winter sports athletes are concerned. In addition, these findings have also provided overall support for the association between motivation and mental disorders and expanded the sequence of the HMIEM. For athletes, mental health is as important as competitive performance, and this research on motivational processes plays an important role in the study of athletes' mental health, both on and off the playing field. Based on these findings, it is essential to consider the various factors in motivational processes of athletes when their mental health status changes.

In this study, mental disorders were found to be positively associated with amotivation and negatively associated with autonomous and controlled motivation, while the negative association between controlled motivation and mental disorders was different from previous studies, perhaps related to the motivational characteristics of winter sports athletes and the behavior of their coaches. It is worth noting that given the relationship between motivational sequences and mental disorders in the HMIEM, the coaching style may have a positive impact on enhancing athletes' motivation and psychological well-being in regard to training and competition. In addition, the findings of this study could make a theoretical contribution to the psychological monitoring and treatment of college and professional sports teams as a whole.

### Limitations

There are several limitations of this research. The first limitation was the location and the structure of the sample. The specific location and sample of the research may limit the generalization of the research results. Therefore, the results of this study may depend on the characteristics of college winter sports athletes in China and may not be generalized to others. The second limitation was the research method. This study was limited by the cross-sectional design through which causal effects could not be determined. Specifically, the current study captured the preferred factors of motivation and mental health for specific groups in a certain period of time. Therefore, a longitudinal study is necessary for future research. The final limitation is the use of correlation. Correlation is a research method that shows the relationship between variables and is not as strict as experimental design (Creswell and Creswell, 2017). Especially for research on athletes' mental health, self-report measures may be biased. Considering causality may require further research, future studies can be supplemented with other methods, such as interviews and experiments.

## Conclusion

To the author's best knowledge, this is the first study to examining the depression, anxiety, stress, and burnout of winter sports athletes within HMIEM, thus providing novel practical implications and addressing a gap in the literature. The sequence in this model shows the associations between motivational factors of HMIEM and mental disorders. Therefore, this research expands earlier investigations in sports psychology. More specifically, a task-oriented climate showed a positive association with basic psychological needs, whereas an ego-oriented climate was negatively associated with basic psychological needs. Basic psychological needs showed a positive association with autonomous and controlled motivation, while there was a negative association with amotivation. Psychological distress and burnout were negatively associated with autonomous and controlled motivation, while being positively associated with amotivation. Autonomous motivation has a greater effect on mental disorders than controlled motivation, while amotivation has the greatest effect. In conclusion, this study shows a relationship between various motivational factors, psychological distress, and burnout among winter sport athletes and provides substantial support for adding mental health factors to the outcomes of the HMIEM sequence.

## Data Availability Statement

The original contributions presented in the study are included in the article/supplementary material, further inquiries can be directed to the corresponding author/s.

## Ethics Statement

The studies involving human participants were reviewed and approved by University of Malaya. The patients/participants provided their written informed consent to participate in this study.

## Author Contributions

XW: conceptualization, formal analysis, data curation, and writing-original draft preparation. XW, NZ, and RA: methodology, validation, resources, writing-review and editing, and project administration. NZ and RA: supervision. All authors have read and agreed to the published version of the manuscript.

## Conflict of Interest

The authors declare that the research was conducted in the absence of any commercial or financial relationships that could be construed as a potential conflict of interest.

## Publisher's Note

All claims expressed in this article are solely those of the authors and do not necessarily represent those of their affiliated organizations, or those of the publisher, the editors and the reviewers. Any product that may be evaluated in this article, or claim that may be made by its manufacturer, is not guaranteed or endorsed by the publisher.
